# The Effects of *Echinacea* (EP107^TM^) on Anxiety: A Comparison of Anxiety Measures in a Randomized, Double Blind, Placebo-Controlled Study

**DOI:** 10.3390/ph18020264

**Published:** 2025-02-17

**Authors:** József Haller, Gábor Faludi, Gábor Kovacs, György Purebl, Zoltán Füzesi, Tamás F. Freund

**Affiliations:** 1 Drug Research Institute, 1083 Budapest, Hungary; 2 Mathias Corvinus Collegium, 1113 Budapest, Hungary; 3 Istvan Nemeskurty Faculty of Teacher Training, Ludovika University of Public Service, 1083 Budapest, Hungary; 4 Department of Psychiatry and Psychotherapy, Faculty of General Medicine, Semmelweis University, 1085 Budapest, Hungary; 5 Department of Psychiatry, State Health Centre, 1097 Budapest, Hungary; 6 Institute of Behavioural Sciences, Faculty of General Medicine, Semmelweis University, 1085 Budapest, Hungary; 7 Anxiofit Ltd., 1033 Budapest, Hungary; 8 Hungarian Academy of Sciences, 1051 Budapest, Hungary

**Keywords:** echinacea, anxiety, humans, psychic anxiety, physical anxiety, herbal anxiolytic

## Abstract

**Background/Objectives**: *Echinacea* extracts with unique alkamide profiles (EP107™) have been shown to affect upper respiratory tract infections and reduce anxiety in both animals and humans. However, a recent study found that a similar extract did not reduce anxiety more than a placebo, although it did enhance well-being and produced antidepressant-like effects. We hypothesized that the discrepancy arose from the differences in the anxiety assessment methods used. The study that observed no effects used the Clinically Useful Anxiety Outcome Scale, which focuses on physical symptoms, while earlier studies used the State-Trait Anxiety Inventory, which focuses on psychic symptoms. **Methods**: To investigate the influence of the anxiety measure on the detectability of anxiolytic effects, we examined the effects of *Echinacea* EP107^TM^ using the Hospital Anxiety and Depression Scale–anxiety subscale (HADS-A), which focuses on psychic symptoms, and the Hamilton Anxiety Rating Scale (HAM-A), most items of which involve physical symptoms. The study was placebo-controlled, double-blind, and multicenter. **Results**: The extract significantly alleviated anxiety compared to placebo when measured with HADS-A. HAM-A total scores did not show significant treatment effects. However, *Echinacea* was superior to placebo in three psychic anxiety items on the HAM-A. **Conclusions**: These findings suggest that *Echinacea* EP107TM reduces psychic anxiety without affecting somatic symptoms. This indicates that the extract may be useful in mild or early-phase anxiety when somatic symptoms are not prominent.

## 1. Introduction

A variety of *Echinacea* preparations have been shown to effectively treat upper respiratory tract infections [1]. Evidence suggests that one particular *Echinacea angustifolia* preparation, identified with the in-house code EP107™, also has psychotropic effects. Specifically, this extract has been found to reduce anxiety in both laboratory animals and humans [2,3,4]. However, a recent study reported antidepressant and well-being-enhancing effects for the same preparation at similar doses, but no impact on anxiety [5]. The reason for this discrepancy remains unknown.

*Echinacea* EP107™ is a blend of hydroalcoholic *Echinacea angustifolia* root extracts obtained from ten different growing sites. The examined herbal formulation did not contain other active ingredients. The extract is standardized for both Echinacoside content and alkamide fingerprint, the latter being the main active ingredient (see below). In all the studies referred to in this publication regarding *Echinacea* effects, this specific extract, produced by one and the same manufacturer, was used. It has been marketed in the US for more than 10 years (https://www.terrynaturallyvitamins.com/anxiocalm); accessed on 3 January 2025. For more details, see Section 4.

**Figure 1:**
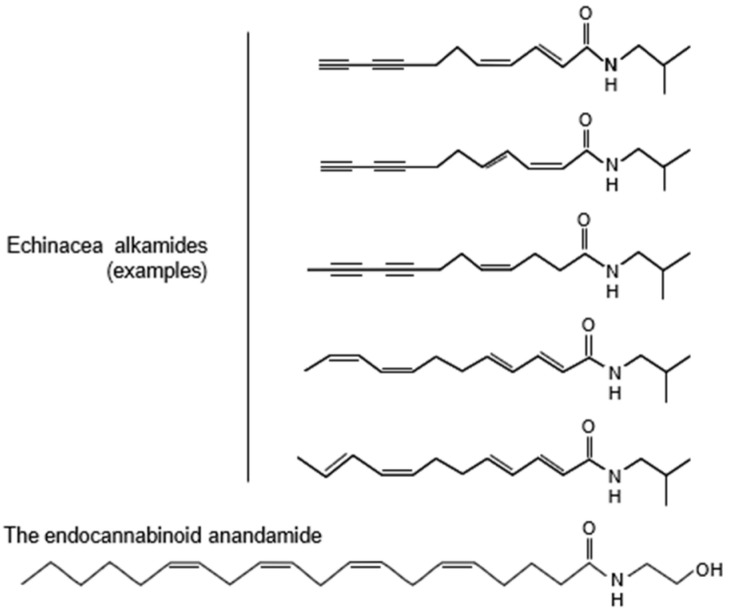
Structural similarities between the endocannabinoid anandamide and *Echinacea* alkamides. Examples of *Echinacea* alkamides are shown; all *Echinacea* alkamides have highly similar structures. For more information on the alkamide structure and their presence in various plants, see Boonen et al. 2012 [12].

The psychotropic effects of *Echinacea* EP107™ may be explained by the alkamides they contain, which affect cannabinoid signaling involved in controlling anxiety and depression [6,7,8,9]. *Echinacea* alkamides are markedly similar to the endocannabinoid anandamide (Figure 1) and bind to the brain CB1 cannabinoid receptor. They also inhibit the fatty acid amide hydrolase (FAAH) enzyme, which degrades anandamide [10]. Isolated alkamides were studied in [35S]GTPγS binding experiments involving rat brain membrane preparations [11]. Significant inverse agonist effects were detected with certain alkamides, while others had partial agonist effects. *Echinacea* alkamides also interacted with the effects of the CB1 agonist reference compound arachidonyl-2′-chloroethylamide. Collectively, these molecular findings show that *Echinacea* alkamides affect endocannabinoid signaling.

In electrophysiological studies, *Echinacea* EP107™ suppressed excitatory synaptic transmission in hippocampal slices without changing inhibitory synaptic transmission [13]. This extract also reduced the spiking activity of CA1 pyramidal cells at doses compatible with brain levels reached after oral administration [14]. Since the hippocampus is involved in controlling anxiety, and anxiety disorders may result from hippocampal hyperactivity [15,16,17], the reduction in hippocampal excitatory synaptic transmission by *Echinacea* is consistent with an anxiolytic effect.

Anxiolytic effects were directly studied through behavioral pharmacological methods in rodents. The same extract that affected molecular mechanisms in the hippocampus reduced anxiety-like behavior in four anxiety tests. It increased open-arm exploration and social interactions in the elevated plus-maze and social interaction tests, respectively, and reduced stress-induced social avoidance and conditioned fear [2,3]. The effective dose range was surprisingly low (3–8 mg/kg) and comparable to that of the benzodiazepine chlordiazepoxide and the selective serotonin reuptake inhibitor fluoxetine, which were tested as comparators in the same studies [2,3].

The anxiolytic potential of *Echinacea* EP107™ was also investigated in human studies using the same extract as in the molecular, electrophysiological, and behavioral pharmacological studies mentioned above. Importantly, the anxiolytic potential was not a general property of *Echinacea* extracts; most were ineffective in laboratory tests [2]. In an early study comparing five different extracts, only one showed anxiolytic potential. Unpublished studies of 12 additional extracts found just one other with similar potential. It was established that the anxiolytic potential of *Echinacea* preparations depends on their specific alkamide fingerprint. A proprietary standardization process involving over ten *Echinacea* production sites ensured the stability of this fingerprint. The in-house code for this fingerprint is EP107^TM^, first used in a publication by Lopresti and Smith [5].

Two of the three published human studies—a dose-control study and a double-blind, placebo-controlled study—used the Spielberger State-Trait Anxiety Inventory (STAI) [18] and reported a significant anxiolytic effect within a few days [3,4]. In the study by Lopresti and Smith [5], anxiety was evaluated using the Clinically Useful Anxiety Outcome Scale (CUXOS) [19]. This study found that anxiety decreased similarly in both the placebo and *Echinacea* EP107™ groups. However, antidepressant-like and well-being-enhancing effects were observed with tests not used in earlier studies, particularly the Positive and Negative Affect Schedule and the Short Form-36 Health Survey.

It has been repeatedly shown that the performance of anxiety screening methods varies and depends on various factors [20,21]. Findings show that the STAI detects anxiolytic effects more readily than other tests, such as the Hamilton Anxiety Rating Scale (HAM-A) or the visual analog scale for anxiety [22,23].

To further test the putative anxiolytic effects of *Echinacea* EP107™, we investigated its effects on human subjects using inventories not previously employed for this purpose. Specifically, we used the Hospital Anxiety and Depression Scale–Anxiety Subscale (HADS-A) [24] and the Hamilton Anxiety Rating Scale (HAM-A) [25]. The items of the former are exclusively related to psychic signs, whereas the latter focuses mainly on physical signs of anxiety. The study was randomized, placebo-controlled, double-blind, and multicenter.

## 2. Results

### 2.1. Patient Characteristics

All patients were Caucasian, aged between 24 and 59 years. Baseline psychometric scores are summarized in Table 1. In total, 15 female patients (across both groups) were of childbearing potential; all urine pregnancy tests were negative. None of the participants used drugs from the following classes: amphetamines, barbiturates, benzodiazepines, cannabinoids, cocaine, opiates, and phencyclidine. Drug tests were negative throughout the study. No abnormalities were detected during the study except for those observed at screening (Table 1).

### 2.2. Effects on Anxiety

**Figure 2:**
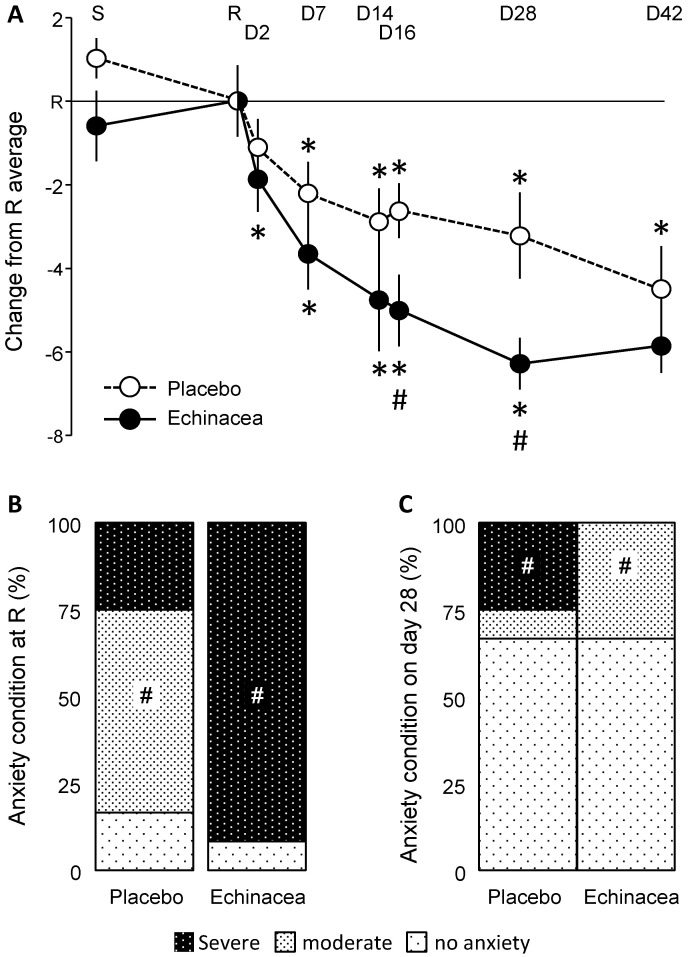
The impact of *Echinacea* EP107™ on HADS-A scores. (**A**) HADS-A scores over the experimental period. Both treatments reduced anxiety compared to the randomization visit, though the effects of *Echinacea* were faster and more pronounced. Notably, ANCOVA was performed on raw data; differences from the randomization visit are shown for clarity. (**B**,**C**) Anxiety state of participants on the randomization day and on the 28th day of the treatment. Anxiety states were determined using established cutoff scores (see Methods). For further explanations, see the text. *D*, study day; *R*, randomization visit; *S*, screening visit; #, significant *Echinacea*/placebo difference; *, significant difference from randomization visit within the same group (*p* < 0.05 at least).

HADS-A scores depended on the interaction between factors (Wilk’s lambda = 0.419; F_interaction_ (7,14) = 2.77; *p* < 0.05). Significant decreases in HADS-A scores were observed on day 2 and day 7 in the *Echinacea* and placebo groups, respectively (Figure 2A). For clarity, data were presented as differences from the day of randomization, but statistical analyses were conducted on raw data. A significant group difference emerged on days 16 and 28, with Hedges’ g values of 0.899 and 1.034, respectively. These values fall into the “large” range based on common benchmarks for Hedges’s g.

The frequency distribution of anxiety states was similar at the screening visit (χ^2^ = 7.64; *p* > 0.1). However, at randomization, the two groups diverged (χ^2^ = 11.90; *p* < 0.036) (Figure 2B). In the placebo group, most participants exhibited moderate anxiety, whereas severe anxiety predominated in the *Echinacea* group. This pre-treatment difference was consistent with the temporal changes observed in HADS-A scores (see above). Despite the pre-treatment dominance of severe anxiety, this state disappeared on the 28th day of treatment in the *Echinacea* group but not in the placebo group (Figure 2C). At this time, 25% of participants still showed severe anxiety in the placebo group, with another 8% exhibiting moderate anxiety. In contrast, no severe anxiety was observed in the *Echinacea* group; those who did not recover completely demonstrated moderate anxiety. By day 42, severe anxiety disappeared from both groups, and most patients’ HADS-A scores fell below the anxiety threshold (χ^2^ = 1.35; *p* > 0.4). Overall, *Echinacea* treatment decreased anxiety more rapidly and effectively than placebo, although the latter had strong effects as well.

**Figure 3:**
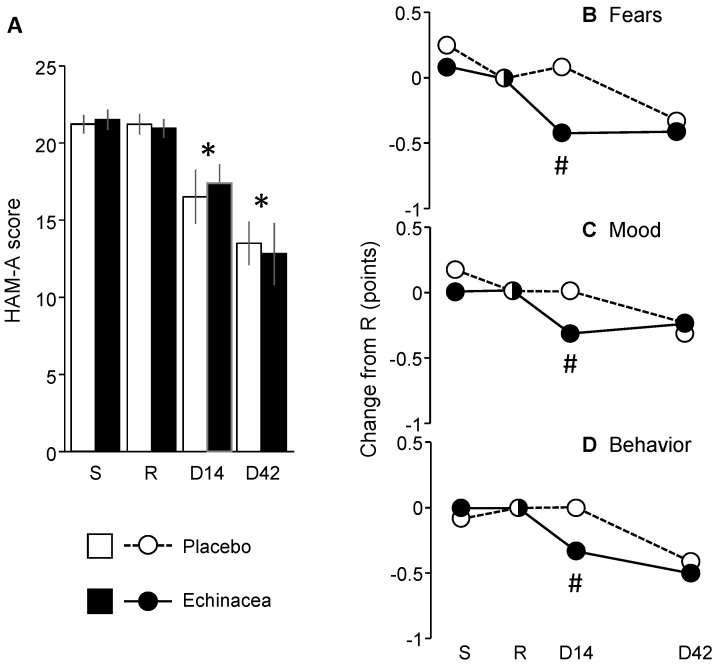
The impact of *Echinacea* EP107™ on HAM-A scores. (**A**) HAM-A scores over the treatment period. (**B**–**D**) Exploratory analyses revealed that three psychic anxiety items of the HAM-A showed larger improvements in the EP107^TM^-*Echinacea* group compared to the placebo group. *D*, day of the study; *R*, randomization visit; *S*, screening visit; #, significant *Echinacea*/placebo difference; *, within-group significant difference from randomization visit (*p* < 0.05 at least).

HAM-A scores remained unchanged during the pre-treatment period but decreased significantly afterward, regardless of the treatment received (Figure 3A) (F_group_ (1,22) = 0.01; *p* > 0.9; F_time_ (3,66) = 36.27; *p* < 0.0001; F_interaction_ (3,66) = 0.41; *p* > 0.7). Scores were consistent with moderate anxiety at screening and randomization visits and with mild anxiety by the end of the treatment period. Exploratory analyses showed, however, that three HAM-A items underwent treatment-dependent changes over time (Figure 3B–D). Specifically, the scores for fears (item 3), depressed mood (item 6), and behavior at interview (item 14) were more favorably affected by *Echinacea* than by placebo (fears: H (1, N = 24) = 5.63; *p* < 0.01; depressed mood: H (1, N = 24) = 4.83; *p* < 0.05; behavior at interview: H (1, N = 24) = 4.73; *p* < 0.05). Therefore, while the overall decrease in HAM-A scores did not depend on the treatment, some individual anxiety items improved more quickly in the EP107^TM^-*Echinacea* group compared to the placebo group.

CGI scores decreased from around 4 (placebo, randomization visit: 4.15 ± 0.10; *Echinacea*, randomization visit: 4.08 ± 0.14) to around 3 by the end of the study (placebo, day 28: 2.92 ± 0.24; *Echinacea*, day 28: 2.77 ± 0.34). This indicates that the experimenter-estimated clinical state of participants improved from moderately ill to mildly ill, but the improvement was independent of treatment (F_treatment_ (1,94) = 0.02; *p* < 0.9; F_time_ (3,94) = 16.96; *p* < 0.001; F_interaction_ (3,94) = 0.15; *p* < 0.9). PSS scores did not show significant changes.

### 2.3. Adverse Events

Two patients were withdrawn from the study, one from the placebo group and the other from the *Echinacea* EP107™ group. Four patients reported nine adverse effects in total; two patients were on placebo, and the other two were on *Echinacea* EP107™. Regarding severity, all events were assessed as mild except one, which was considered moderate intensity. This moderate event was a depressive mood reported by a patient in the placebo group. None of the adverse events required any treatment, and all resolved spontaneously. Concerning physical examinations, five patients had abnormal physical findings (e.g., hypertension): two were from the *Echinacea* EP107™ group, and three were from the placebo group. Therefore, the frequency of adverse events was similar in both the placebo and *Echinacea* EP107™ groups.

## 3. Discussion

### 3.1. Main Findings

*Echinacea* EP107™ reduced anxiety more effectively than the placebo according to HADS-A scores. The anxiolytic effects were observed more rapidly and were stronger than those of the placebo. However, HAM-A scores decreased regardless of the treatment received, probably because focusing heavily on physical symptoms obscured potential effects on other aspects of anxiety. In line with this, *Echinacea* had a stronger effect on three specific HAM-A psychic anxiety items compared to the placebo. The incidence and severity of adverse events were similar in both treatment groups.

### 3.2. Overall Interpretation

The placebo effects observed in this study were unexpectedly strong. In a previous placebo-controlled, double-blind study, the placebo effects were considerably weaker and only transient. We hypothesize that the strong placebo effects in the current study were due to the conditions under which it was conducted. It has been demonstrated that placebo effects increase when patients perceive the healing environment as optimal, site visits are frequent, and patient expectations are high due to a meaningful doctor–patient relationship [26,27,28,29]. In the study by Haller et al. [4], visits were less frequent, and the study site was a health center for various diseases, not limited to psychiatric disorders. In contrast, the current study was conducted in leading psychiatric clinics or psychiatric departments in Budapest. The investigators were well-known academics, including professors from Semmelweis University, one of Hungary’s most prestigious medical universities. This environment may account for the differences in placebo effects observed in the present and the earlier study.

Meta-analyses suggest that, in psychiatric disorders, the placebo effect is nearly as significant as, and contributes substantially to, the effect of active medications [29,30]. Some authors suggest that placebo effects should be harnessed in clinical practice by creating an optimal treatment environment that enhances them [28,29]. However, in a research context, placebo effects can obscure the true effects of medications. In our study, *Echinacea* EP107™ was superior to the placebo according to HADS-A scores, suggesting its potential as an active medication for anxiety.

However, *Echinacea* did not outperform the placebo in the case of HAM-A, a standard measure of anxiety. Several hypotheses can be formulated regarding this discrepancy. Considering the variability in the ability of different tests to detect the anxiolytic effects of agents [20,21,22,23], it can be hypothesized that the State-Trait Anxiety Inventory and HADS-A are effective in assessing these effects, whereas HAM-A and CUXOS are not appropriate for testing *Echinacea*’s anxiolytic properties. This is supported by this and earlier studies. Another hypothesis suggests that the placebo alone may have eliminated HAM-A anxiety, preventing further improvement by active treatment. Supporting this assumption, the study by Lopresti and Smith [5] found that the placebo decreased CUXOS scores below the cutoff for anxiety. If anxiety is abolished, it cannot be further improved. Additionally, positive findings with HADS-A may be attributed to it being a self-assessment tool that could be administered more frequently, enabling early detection of effects when the placebo alone had not yet reduced anxiety.

An alternative explanation is that *Echinacea* EP107™ was effective against psychic but ineffective against somatic anxiety symptoms. Lopresti and Smith [5] previously noted this possibility when comparing their findings with those of Haller et al. [4]. They highlighted that all 40 items of the STAI assess psychic anxiety, whereas 70% of CUXOS items investigate somatic anxiety. A similar difference exists between HADS-A and HAM-A; the former exclusively examines psychic anxiety, whereas most items of the latter address somatic complaints resulting from anxious feelings. As such, it can be hypothesized that *Echinacea* EP107™ is effective against psychic anxiety but not somatic symptoms. This is further supported by the positive effects of *Echinacea* on three psychic items of HAM-A.

### 3.3. Putative Mechanisms of Action

Earlier studies have shown that *Echinacea* alkamides bind to and affect the function of CB1 receptors and enhance anandamide signaling by inhibiting its degradation by the FAAH enzyme [10,11]. Unidentified *Echinacea* constituents—possibly alkamides—are also agonists of TRPV1 receptors [31]. These effects may be relevant to the anxiolytic properties of *Echinacea* because the CB1 receptor plays a significant role in the control of anxiety and is a current target for the development of novel medications for mental disorders, including anxiety [7,32]. FAAH inhibitors are also targets for the development of anxiolytic medications [33,34], and TRPV1 receptor ligands are considered promising for the development of novel anxiolytics [35,36].

The experimental findings briefly reviewed here do not clarify the mechanism of action of *Echinacea*, particularly why one extract, such as *Echinacea* EP107™, is more effective than others. However, they do show that *Echinacea* alkamides influence brain mechanisms that play a role in anxiety. Further research is necessary to map out these mechanisms in detail.

### 3.4. Limitations

The two main limitations of this study are related to sample size and the large placebo effect. The necessary sample size was calculated based on accepted principles (see above) and was sufficient to detect significant changes over the trial. However, it was smaller than those generally used in studies on the efficacy of anxiolytics, and additional trials with larger study populations are needed to validate these findings. The placebo effects observed were surprisingly strong in this study. By the end of the treatment period, anxiety had substantially decreased in both groups, with similar anxiety scores in the placebo and *Echinacea* groups by day 42. Longer-term follow-up would be beneficial to assess the duration and sustainability of any observed effects. This could be examined by studying the decay of effect after washout periods of varying lengths. Further clinical research on this formulation, focusing on its anxiolytic effect, is required.

## 4. Materials and Methods

### 4.1. Participants

We studied 26 middle-aged subjects (43.3 ± 1.9 years); 7 were males and 19 were females. The gender ratio matched the European gender distribution of anxiety disorders [37]. Although the investigational product had been used in the US for several years, there were no previous human data available for this particular indication to support a formal sample size calculation. Therefore, a sample size of 12 participants per group was chosen based on feasibility, precision about the mean and variance, and regulatory considerations as described by Julious [38]. We screened 27 potential participants to account for potential dropouts.

The study was approved by the National Institute of Pharmacy (Budapest, Hungary) under registration number OGYI/23775-6/2010. It was conducted in accordance with the Declaration of Helsinki, applicable Hungarian legislation on human studies and medical data protection, and Good Clinical Practice guidelines. This study was registered in the EU Clinical Trials Register (EudraCT Number: 2010-020431-33, Protocol Number: ANX001).

### 4.2. Inclusion Criteria

Participants were 18 to 60 years old (inclusive) with a diagnosis of generalized anxiety disorder (GAD) according to DSM-IV criteria at the screening visit. They had a total HAM-A score between 17 and 25 points at screening, a total Beck Depression Inventory (BDI) score lower than 10, and were in good physical health, defined by no clinically relevant symptoms identified through detailed medical history and a full physical examination including sitting blood pressure and heart rate measurement. Participants were required to sign an informed consent form indicating their ability and willingness to comply with study procedures.

### 4.3. Exclusion Criteria

Participants were excluded if they had any DSM-IV Axis I diagnosis within the 6 months preceding the study, showed any Axis II disorder, presented a serious suicidal risk, or currently used psychotropic medications that could not be discontinued before randomization (3 months for benzodiazepines, 5 weeks for fluoxetine, and 14 days for monoamine oxidase inhibitors). Participants were also excluded if they currently used drugs, supplements, prescription or nonprescription, or foods with psychoactive properties, were subjects of formal psychotherapy within 3 months before screening, had positive drug tests at screening or randomization for certain drugs, had a history of allergies or intolerance to any *Echinacea* product, or were treated with *Echinacea* within 60 days before the first dose of trial medication. Women had to be non-lactating, non-pregnant (checked by pregnancy tests), and using hormonal or barrier contraception or be postmenopausal.

### 4.4. Study Design and Treatments

This was a randomized, double-blind, parallel-group, multi-site, placebo-controlled, fixed-dose Phase 2 study involving *Echinacea* EP107™ and placebo in outpatients with generalized anxiety disorder. It was conducted under the management of Accelsiors CRO (https://accelsiors.com, accessed on 3 January 2025).

Treatments consisted of 7 mm diameter, round, biconvex tablets administered twice daily (morning and evening). The tablets either contained the proprietary *Echinacea* EP107™ extract with a unique alkamide profile (20 mg per tablet, 40 mg per day) or its excipients only (placebo). Blinding was maintained with identical film-coated tablets, with emergency code envelopes available for unblinding if needed. Both types of tablets were manufactured by ExtractumPharma Co (Budapest, Hungary) and registered by the National Institute for Food and Nutrition Science (file No. 2249-4/2010 OÉTI).

### 4.5. Investigators and Sites

The investigators were trained psychiatrists from the following four clinics: Bajcsy-Zsilinszky Hospital (Department of Psychiatry), Normental Medical and Organizational Limited Partnership Company, Semmelweis University (Institute of Behavioral Sciences and the Clinic of Psychiatry and Psychotherapy), and the State Health Center (Department of Psychiatry). The study was conducted from late summer to winter (August–February).

### 4.6. Study Procedures

**Figure 4:**
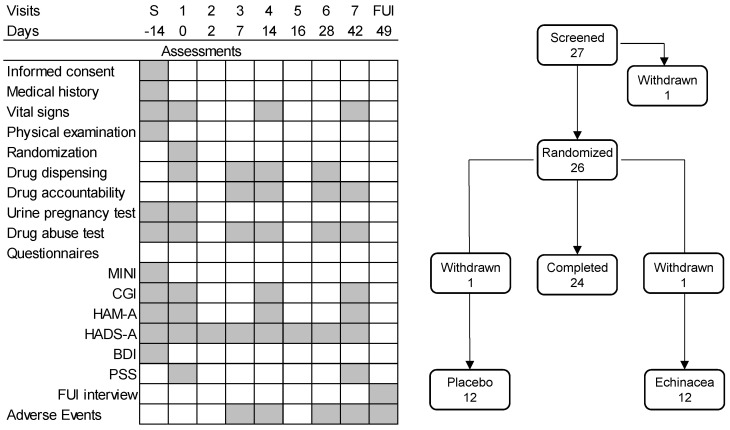
The design of the study. Left-hand panel: timing of assessments. Right-hand panel: number of participants over the phases of the study. Abbreviations: BDI, Beck Depression Inventory; CGI, Clinical Global Impression; FUI, follow-up interview; HADS-A, Hospital Anxiety and Depression Scale–Anxiety subscale; HAM-A, Hamilton Anxiety Rating Scale; MINI, MINI-International Neuro-psychiatric Interview; PSS, Perceived Stress Scale. ‘Withdrawn’ denotes participants who withdrew consent to participate in the study. Note that the HADS-A is a self-report instrument, administered repeatedly, while the HAM-A is a clinician-administered instrument, completed only during visits to the study sites. The validity of home-administered HADS-A assessments was studied on study days R and 2, as well as days 14 and 16, where the same inventory was completed both at the clinic and at home at two-day intervals.

The study design is shown in Figure 4. New patients arriving at the study sites were informed about the study in detail and were offered the opportunity to participate. Those willing to participate signed an informed consent form, after which they underwent a medical and psychiatric examination. The medical history of participants was checked, followed by a physical examination and psychometric tests, as shown in Figure 4. Those who met the criteria listed above were asked to return after two weeks to start the study.

The six-week treatment phase began with the randomization visit when participants were examined again, randomized to *Echinacea* EP107™ or placebo treatments, and received the examined herbal formulation in the form of identical white tablets. Treatments started on the randomization day. Thereafter, drugs were dispensed on days 1, 7, 14, and 28 of the study. Treatment compliance was checked each time, and participants returned unused tablets. At each visit, participants underwent psychiatric testing as shown in Figure 4. Vital signs were checked at screening, randomization, and on days 14 and 42. Drug abuse was checked at each study site visit. Urine pregnancy tests were performed at screening and randomization, and during subsequent visits, if participants reported the possibility of pregnancy. Adverse events were checked on days 7, 14, 28, and 42. The study ended on day 49 with a follow-up interview focusing on adverse effects and the potential need for further therapeutic interventions.

Participants completed the Hospital Anxiety and Depression Scale–Anxiety subscale (HADS-A) at home beginning on the second day of treatment (Figure 4). To validate home scoring, assessments followed clinical screening by two days on days 2 and 16 of the study. Scores were highly similar, demonstrating the reliability of home scoring. Structured self-assessment diary techniques like these have been used in various disorders, including anxiety [39,40].

### 4.7. Psychometric Instruments

Diagnosis was established using the structured MINI-International Neuropsychiatric Interview [41]. Note that the version of the MINI we used was based on DSM-IV. Nevertheless, the description of GAD did not change significantly from DSM-IV to DSM-5; consequently, the MINI diagnosis remained valid. Anxiety was assessed by the HADS-A and Hamilton Anxiety Rating Scale (HAM-A). Depression symptoms and life events were evaluated using the Beck Depression Inventory (BDI) [42]. Stress perception was measured using the Perceived Stress Scale (PSS). Additionally, anxiety severity was evaluated using the Clinical Global Impression scale (CGI).

HADS-A: A self-report questionnaire designed to evaluate the severity of anxiety symptoms [43]. It consists of 7 items scored on a 4-point scale from 0 (not present) to 3 (considerable). Item scores are summed, yielding total scores from 0 to 21. Cutoff scores for normal, moderate, and severe anxiety are 0–7, 8–10, and 11–21, respectively.

HAM-A: A clinician-administered scale measuring the severity of anxiety symptoms [44,45]. It consists of 14 items, each defined by a series of symptoms. Cutoff scores for normal, mild, moderate, and severe anxiety are 0–9, 10–15, 16–24, and 25–42, respectively.

BDI: Identifies the presence and severity of symptoms consistent with depression criteria in the DSM-IV. Used only for eligibility assessment during the screening visit.

PSS: Measures the degree to which life situations are perceived as stressful [46]. The 10-item version was employed. No cutoff scores are available for this test.

CGI: A 7-point scale on which the clinician rates the severity of the patient’s illness relative to personal experience with patients with similar diagnoses [47]. Ratings are: 1 (not ill) to 7 (extremely ill).

We used the validated Hungarian versions of all tests: MINI [48]; HADS-A [49]; HAM-A [50]; BDI [51]; PSS [52].

### 4.8. Other Measures

Vital signs were evaluated after at least 5 min of rest at screening and randomization: body temperature, sitting blood pressure, respiratory rate, and pulse rate. A trained physician evaluated physical condition, including an external assessment of the head, eyes, ears, nose, throat, lungs, cardiovascular system, breast, abdomen, musculoskeletal system, skin, lymph nodes, and central nervous system (e.g., Achilles reflex). Body weight was also recorded. Physical condition was checked again on the last day of the study. Pregnancy, an exclusion criterion, was evaluated via a clinical urine test at the local lab. Suspected pregnancies during the study were checked similarly. Drug abuse was evaluated by routine laboratory tests for the following drug classes: amphetamines, barbiturates, benzodiazepines, cannabinoids, cocaine, opiates, and phencyclidine.

Adverse events, i.e., any undesirable signs, symptoms, or medical conditions that occurred after starting treatment, were recorded even if not considered related to treatment. Such events could be reported by participants, discovered by investigators, or detected through physical examination, laboratory tests, or other means. Serious adverse events would have led to treatment discontinuation until they resolved. No such instances occurred during the study. Safety assessments included monitoring and recording adverse events, vital signs, physical condition, and body weight changes.

### 4.9. Statistics

The main objective was to conduct a preliminary evaluation of the safety and efficacy of *Echinacea* EP107™ compared to placebo for treating generalized anxiety disorder and to provide a variance estimate for formal sample size calculations in future studies. Sample sizes were described above, and statistics were performed on the per the protocol subset (PP).

Data are presented as the mean ± standard error of the mean (SEM), except for categorical data, which are shown as ratios (e.g., male/female ratio) and compared using crosstabulation. Psychometric scores were evaluated using a two-factor repeated measures ANOVA, with time (visits as levels) as the repeated measures factor and treatment (levels: *Echinacea* and placebo) as the second factor.

Unexpectedly, there was a significant change in HADS-A scores between screening and randomization (Figure 2A). Specifically, HADS-A scores increased in the *Echinacea* group and slightly decreased in the placebo group. To account for this pre-treatment difference, HADS-A data were analyzed using repeated measures ANCOVA, with treatment and time as categorical factors and the randomization day as the continuous predictor. The Duncan test was used for post-hoc comparisons. Discrete data of a narrow range (e.g., individual items of the HAM-A) were compared using Kruskal–Wallis ANOVA. Effect sizes were evaluated by calculating Hedges’ g. A value of *p* < 0.05 was considered significant.

## 5. Conclusions

We found that *Echinacea* EP107™ reduced anxiety more effectively than the placebo. This effect developed against a backdrop of a favorable side-effect profile. The inconsistencies between anxiety tests can be attributed to *Echinacea*’s primarily psychic anxiety-reducing effect. This suggests that the preparation is effective in mild forms or the early stages of anxiety, where somatic symptoms are not yet pronounced.

Anxiety disorders, as well as anxieties associated with somatic illnesses, often exhibit a variable course [53,54,55,56,57]. It has been proposed that patients experiencing fluctuating anxiety levels might benefit from fast-acting therapy, particularly with benzodiazepines [7,10,31,32,33,34,35,36,53,54,55,58,59]. However, a significant proportion of individuals with psychiatric disorders, especially those experiencing depression and anxiety, choose alternative therapies over conventional treatments [60,61,62]. The motivations for choosing non-conventional approaches vary, including personal beliefs about maintaining a healthy lifestyle and concerns about the potential side effects of standard medical treatments [60,61,62,63]. Our study indicates that the combined demand for fast-acting anxiolytics and preference for non-conventional therapies can be addressed through the E. *angustifolia* extract evaluated in this and previous research.

## Figures and Tables

**Table 1:** Patient characteristics.

Variable	Placebo	*Echinacea*	Statistics
Gender ratio (male/female)	3/10	4/9	χ^2^ = 0.20 *p* > 0.6
Prior diseases (T/nT)	6/7	8/5	χ^2^ = 0.62 *p* > 0.4
Prior psychotropic medication (yes/no)	1/12	2/11	χ^2^ = 0.38 *p* > 0.5
Physical abnormality * (yes/no)	3/10	2/11	χ^2^ = 0.25 *p* > 0.6
HADS-A	10.5 ±0.5	11.3 ±0.8	F(1,24) = 0.76 *p* > 0.3
HAM-A	21.1 ±0.6	21.6 ±0.7	F(1,24) = 0.41 *p* > 0.5
CGI	4.15 ±0.10	4.08 ±0.14	F(1,24) = 0.02 *p* > 0.9
PSS	33.5 ±1.6	31.4 ±1.4	F(1,24) = 1.05 *p* > 0.3
BDI	6.8 ±0.7	5.4 ±0.5	F(1,24) = 2.46 *p* > 0.1

*, Achilles areflexia, gastroesophageal reflux disease, hypertension, lost vision one eye; light vein varicosity on legs. BDI, Back Depression Inventory; CGI, Clinical Global Impression; HADS-A, Hospital Anxiety and Depression scale, anxiety subscale; HAM-A, Hamilton rating scale for anxiety; nT, previously not treated for major disease; PSS, Perceived Stress Scale; T, previously treated for major disease.

## Data Availability

The raw data presented in this study are openly available in Zenodo at https://doi.org/10.5281/zenodo.14610434. Any additional inquiries can be directed to the corresponding author.
